# Supplementary material for: Direct Photochemical Synthesis of Substituted Benzo[b]fluorenes
Source: Org Lett. 2024 Nov 25;26(48):10364–8. doi: 10.1021/acs.orglett.4c03978 (PMC11629382; doi:10.1021/acs.orglett.4c03978)
Supplement: Supplementary file 2 — ol4c03978_si_002.zip [file ol4c03978_si_002.zip › FID for Publication/5b-D1/plots/RC-2-C-5-161F15-21_PROTON_20240927_03_plot01.pdf]

RC-2-C-5-161F15-21

Location	7	Pulse sequence	PROTON	Temperature	25	Suboperator	RuairiCrawford
Date collected	2024-09-27	Solvent	cd3cn	Spectrometer	Agilent-NMR-vnmrs500	Operator	MBaumann

SAMPLE

date	Sep 27 2024
solvent	cd3cn
file	/home/data/MBaumann/newucd500/RuairiCrawford/2024/20240927_RC-2-C-5-161F15-21_01/RC-2-C-5-161F15-21_PROTON_20240927_03.fid

ACQUISITION

sw	8012.8
at	2.600
np	41666
fb	4000
bs	2
ss	2
d1	2.000
nt	16
ct	16

TRANSMITTER

tn	H1
sfrq	500.036
tof	500.0
tpwr	62
pw	3.500

DECOUPLER

dn	C13
dof	0
dm	nnn
decwave	W40_One_NMR_new
dpwr	39
dmf	32258

PRESATURATION

satmode	n
wet	n

SPECIAL

temp	25.0
gain	30
spin	0
hst	0.008
pw90	7.000
alfa	10.000

FLAGS

il	n
in	n
dp	y
hs	nn

PROCESSING

fn	32768

DISPLAY

sp	-1005.7
wp	8012.3
rfl	1006.2
rfp	0
rp	15.9
lp	0

PLOT

wc	252
sc	8
vs	121
th	1
ai cdc ph	

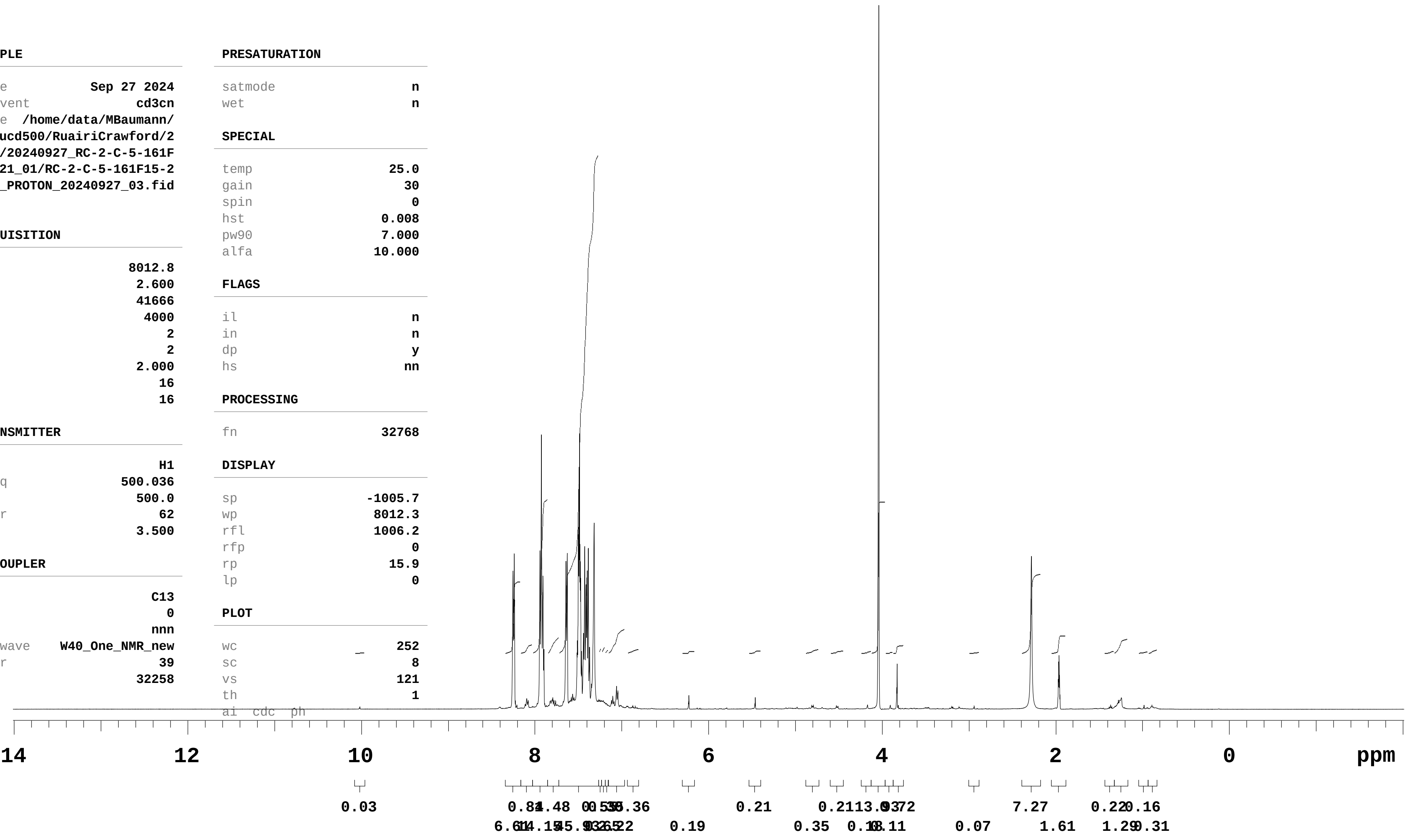

Plotname: RC-2-C-5-161F15-21_PROTON_20240927_03_plot01

Data file /home/data/MBaumann/newucd500/RuairiCrawford/2024/20240927_RC-2-C-5-161F15-21_01/RC-2-C-5-161F15-21_PROTON_20240927_03.fid

Plot date 2024-09-27
